# Quadrate orientation and joint reaction force underwent correlated evolution during suchian evolution

**DOI:** 10.1111/joa.70020

**Published:** 2025-07-22

**Authors:** Kaleb C. Sellers, Alec T. Wilken, Corrine R. Cranor, Kevin M. Middleton, Casey M. Holliday

**Affiliations:** ^1^ Department of Organismal Biology and Anatomy University of Chicago Chicago Illinois USA; ^2^ Department of Pathology and Anatomical Sciences University of Missouri Columbia Missouri USA; ^3^ Division of Biological Sciences University of Missouri Columbia Missouri USA

**Keywords:** biomechanics, crocodylian, jaw joint, joint reaction force, quadrate

## Abstract

As part of the jaw joint, the quadrate is a key skeletal element of the feeding system in nonmammalian vertebrates, which plays a critical role in resisting joint reaction forces (JRF). Some authors have suggested that the quadrate orientation reflects overall muscle anatomy and, by implication, JRF. Here, we quantitatively test the longstanding hypothesis that quadrate orientation is correlated with JRF orientation using the suchian lineage leading to extant crocodylia. The evolution of the characteristic crocodylian skull is a major transformation in vertebrate evolution in which the quadrate played a crucial role. We use detailed, three‐dimensional biomechanical modeling to estimate JRF in a sample of eleven fossil and extant suchians and compare these to the orientation of quadrates. We use the cross‐product of orientation vectors to quantify similarity in orientation and show that the angle of the quadrate in the sagittal plane is tightly coupled with JRF in the same. These results demonstrate a coordinated evolution between JRF and quadrate anatomy during suchian evolution and provide a framework with which to analyze evolutionary changes in joint anatomy and biomechanics.

## Graphical abstract

The quadrate is the cranial bone of the nonmammalian jaw joint. The quadrate has long been hypothesized to mirror the direction of joint reaction forces (JRF) during feeding. This study uses 3D biomechanical modeling to reveal a striking evolutionary link between quadrate orientation and JRF direction, unlocking new insights into the dynamic forces shaping the iconic crocodylian skull.

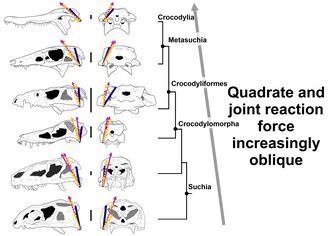

## 1 INTRODUCTION

The jaw joint is a key feature of the feeding system in tetrapods. It facilitates and modulates movement and force transfer between the cranium and mandible, thereby exerting a strong influence on craniomandibular biomechanical performance and evolution. Crucially, the jaw joint withstands all jaw muscle force that is not transmitted as bite force during feeding (Greaves, 1978; Greaves, 1998; Terhune et al., 2022). Both bite forces and their associated jaw joint reaction forces (JRFs) are ecologically relevant because they inform how the feeding system interacts with the environment and with itself, respectively. Although we know much about bite forces (Erickson et al., 2003), we know comparatively little about the reaction forces at the jaw joint. JRFs are often considerably higher than bite forces in tetrapod feeding systems (Crompton & Hylander, 1986), making joint reaction force a major source of cranial loading that impacts morphology, function, and performance of the jaw joint. Thus, characterizing joint reaction force is critical not only for understanding vertebrate feeding functional morphology and biomechanics, but also vertebrate ecology and evolution.

Jaw joint morphology correlates with feeding‐induced loading in numerous tetrapods (Bramble, 1978; Cleuren et al., 1995; Sinclair & Alexander, 1987). For example, the quadrate of nonmammalian tetrapods is well suited to resist compressive forces but less suited for tensile loads (Liu & Herring, 2000). Various authors have qualitatively noted the similar orientation between the quadrate and the jaw elevator muscles in crocodylians, lizards, amphisbaenians, and turtles (Bramble, 1978; Cleuren et al., 1995; Sinclair & Alexander, 1987). These works suggest that the quadrate is oriented to maximize resistance to axial loading and minimize bending loads, regardless of its orientation relative to the rest of the cranium.

**FIGURE 1:**
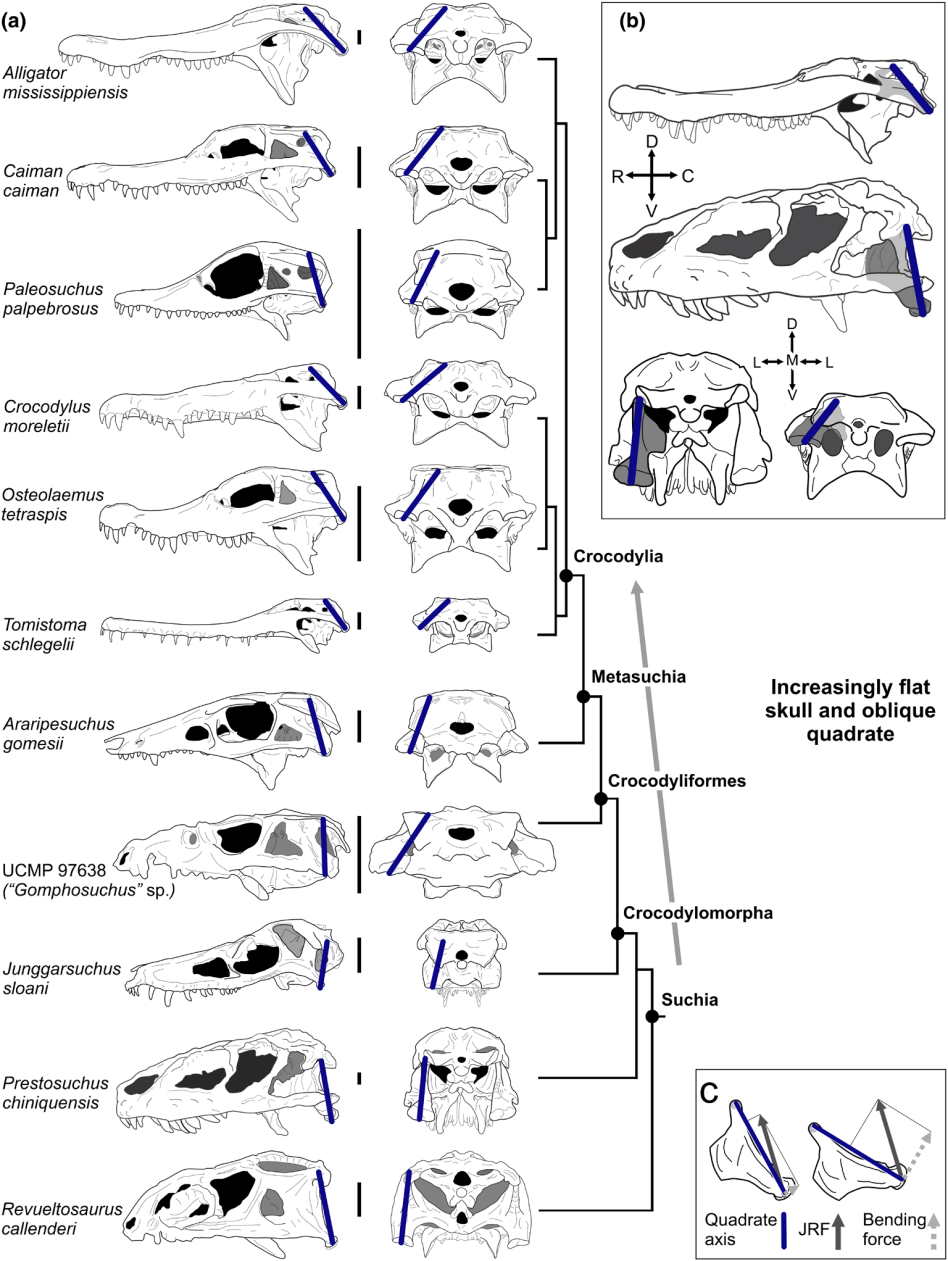
(a) Suchian evolution is characterized by progressive skull flattening and reorientation of the quadrate. (b) Comparison of alligator (top; right) and Prestosuchus (bottom; left) shows the more oblique orientation of the quadrate (gray highlight) in extant crocodylians relative to early suchians. (c) A given joint reaction force can produce different loading regimes depending on the orientation of the quadrate. Blue lines show quadrate axes.

Here, we use the evolutionary transformation of crocodylian‐line archosaurs (suchians) from tall‐skulled terrestrial predators to flat‐skulled aquatic ambushers to quantitatively test this hypothesis with three‐dimensional morphometric and biomechanical data. Throughout their long evolutionary history, suchians explored a wide range of cranial ecomorphologies, ranging from forms adapted to terrestrial predation to herbivory with oral processing to aquatic ambushing common in extant crocodylians. One of the most striking features of suchian evolution is the progressive skull flattening effected in part by a major transformation to the quadrate (Figure 1a). In early suchians, the quadrate was dorsoventrally orientated with relatively weak connections to the skull roof, braincase, and palate (Figure 1b; Clark, 1994; Kuzmin et al., 2021). By contrast, in extant crocodylians, the quadrate is rigidly sutured to the braincase and is obliquely oriented, positioning the jaw joint caudal to the occiput.

In Crocodyliformes, the quadrate reoriented mediolaterally, shifting the primary dorsal contact of the quadrate from the skull roof to the braincase and the quadratoarticular joint caudal to the occiput (Clark, 1994; Kuzmin et al., 2021; Langston, 1973; Tarsitano, 1985). Additionally, its contacts with the palate and braincase became immobilized via extensive suturing (Clark, 1994; Kuzmin et al., 2021; Langston, 1973). In extant crocodylians, this oblique orientation of the quadrate and apomorphically flat skull facilitates aquatic ambush predation (Grigg & Kirshner, 2015; Iordansky, 1973). However, flat skulls impose various constraints on the feeding system and performance. A flat skull has a dorsoventrally compressed adductor chamber, causing jaw muscles to become shortened or to be constrained to mechanically inefficient orientations (Herrel et al., 2006; Sellers et al., 2022). This led to ecological and biomechanical selective pressures acting in opposing directions, making suchians an ideal clade for exploring the relationships between skull shape, muscle and joint anatomy, and feeding biomechanics.

To evaluate the functional implications of jaw joint reorientation, we integrate three‐dimensional jaw muscle data from extant crocodylians with reconstructed musculature from fossil suchians to estimate bite forces and JRFs. As crocodylian skulls became increasingly flattened, jaw muscles reoriented into substantially rostrocaudal and mediolateral orientations (Sellers et al., 2022), and studying JRF in three dimensions is necessary. We then characterize the orientation of the quadrate and compare this with the orientation of JRF to elucidate the relationship between quadrate orientation and joint loading. Using these data, we test the longstanding hypothesis that quadrate orientation corresponds to joint loading. We make three specific predictions:

Hypothesis 1 *The orientation of the quadrate axis and joint load vectors will be more similar than would be expected by chance*.

Hypothesis 2 *Quadrate orientation is more similar to the orientation of higher magnitude loads than lower magnitude loads*.

Hypothesis 3 *JRF orientation can be predicted by the orientation of the quadrate*.

## 2 METHODS

### 2.1 Specimens

Our analysis included six extant crocodylians: Alligator mississippiensis (MUVC 008), Caiman crocodilus (FMNH 73711), Paleosuchus palpebrosus (FMNH 22817), Crocodylus moreletii (TMM M‐4980), Osteolaemus tetraspis (FMNH 98936), and Tomistoma schlegelii (TMM M‐6342). We chose fossil taxa that demonstrate the changes that took place in the lineage leading to the crown group while avoiding highly derived extinct taxa. We acquired jaw muscle anatomy in our extant sample with computed tomography (CT) imaging, contrast‐enhanced CT imaging/diceCT (Gignac et al., 2016; Holliday et al., 2022), and traditional dissections. We also analyzed five extinct suchians that show different degrees of skull flattening and concomitant rotation of the quadrate: Araripesuchus gomesii (AMNH 24450), an unnamed “protosuchian” informally called “Gomphosuchus” sp. (UCMP 97638; Clark, 1986), Junggarsuchus sloani (IVPP V14010), Prestosuchus chiniquensis (UFRGS PV0629T), and Revueltosaurus callenderi (PEFO_34561; Parker et al., 2021). A list of institutional abbreviations may be found in Table 1.

**TABLE 1:** List of institutional abbreviations for species used in the present study.

Institution	Abbreviation
American Museum of Natural History	AMNH
Field Museum of Natural History	FMNH
Institute of Vertebrate Paleontology and Paleoanthropology	IVPP
University of Missouri Zoological Collections	MUVC
Petrified Forest National Park	PEFO
Texas Memorial Museum	TMM
University of California Museum of Paleontology	UCMP
Federal University of Rio Grande do Sul Museum of Paleontology	UFRGS

### 2.2 Muscle modeling

To estimate the magnitude and orientation of muscle forces, we used 3D models of specimens created for a previous study (Sellers et al., 2022). The specimens were scanned either with CT or 3D laser surface scanning. The three‐dimensional structure of the bones was obtained by manually segmenting the scan data using Avizo Lite 9.4 (FEI Visualization Science Group; https://www.thermofisher.com). The models were then processed using Geomagic Design X (Geomagic, Inc.; https://www.3dsystems.com) to clean, smooth, and align them with the global anatomical axes (x for mediolateral, y for dorsoventral, and z for rostrocaudal). The mandibles were opened to a five‐degree gape to minimize joint loading due to low jaw muscle mechanical advantage from high‐gape bites. Subsequently, the models were meshed and filled with tetrahedra in Strand7 (Strand7 Pty. Ltd.; http://www.strand7.com) for subsequent biomechanical modeling. Bone was modeled as an orthotropic material with the following material properties: mediolateral Young's modulus = 8.8 GPa, dorsoventral Young's modulus = 10.65 GPa, rostrocaudal Young's modulus = 20.49 GPa, shear modulus in the axial plane = 3.35 GPa, shear modulus in the horizontal plane = 4.96 GPa, shear modulus in the sagittal plane = 5.92 GPa, mediolateral Poisson's ratio = 0.4, dorsoventral Poisson's ratio = 0.14, and rostrocaudal Poisson's ratio = 0.14 (Zapata et al., 2010). In specimens with joints hypothesized to be mobile, suture or cartilage beams were added. Material properties were as follows: suture: Poisson's ratio = 0.3, Young's modulus = 10 MPa (McLaughlin et al., 2000); cartilage: Poisson's ratio = 0.49, Young's modulus = 6 MPa (Beaupré et al., 2000). Summaries of models may be found in Table S1.

Anatomically detailed muscle attachment sites were determined based on osteological correlates, contrast‐enhanced CT imaging, dissections, and references to the literature (Bona & Desojo, 2011; Busbey, 1989; Holliday & Witmer, 2007; Holliday & Witmer, 2009; Iordansky, 1964; Iordansky, 2000; Schumacher, 1973; Sellers et al., 2017; Sellers et al., 2022). Modeled muscles include: *musculus adductor mandibulae externus superficialis* (mAMES), *musculus adductor mandibulae externus medialis* (mAMEM), *musculus adductor mandibulae externus profundus* (mAMEP), *musculus adductor mandibulae posterior* (mAMP), *musculus pseudotemporalis superficialis* (mPSTs), *musculus pterygoideus dorsalis* (mPTd), *musculus pterygoideus ventralis* (mPTv), and *musculus depressor mandibulae* (mDM). Muscle terminology follows Holliday and Witmer (2007). Muscle attachment sites are shown in Figures S3–S13 and on Sketchfab (Table S2). The calculation of the physiological cross‐sectional area (PCSA) involves combining information about attachment site geometry and muscular parameters, as described in Equation 1 (Sacks & Roy, 1982):

(1) $$\text{PCSA}=\frac{V_{M}}{l_{f}}\cdot \cos\left(\theta \right),$$

where *V*_*M*_ is muscle volume, *l*_*f*_ is relative fascicle length, and *θ* is pennation angle. To estimate muscle volume, we modeled muscle origins and insertions as the two faces of a frustum (Sellers et al., 2017), defined in Equation 2:

(2) $$V_{M}=\frac{l_{f}}{3}\cdot (A_{\text{or}.}+A_{\mathrm{ins}.}+\sqrt{A_{\text{or}.}\cdot A_{\mathrm{ins}.}),}$$

where *A*_or_. is muscle origin area and *A*_ins_. is muscle insertion area. The muscle force (*F*_*M*_) from a given PCSA is mediated by specific tension, defined in Equation 3:

(3) $$F_{M}=\text{PCSA}\cdot T_{\text{specific}},$$

where *F*_*M*_ is muscle force and *T*_specific_ is specific tension. We use a value of 30 N/cm^2^ (Hieronymus, 2006). Muscular parameters that could not be estimated directly from fossil morphology (e.g., relative fascicle length, specific tension, pennation angle.) were given values from *Alligator* (Porro et al., 2011). To characterize muscle orientation, we subtracted the centroid of the insertion from the centroid of the origin to create a muscle vector, as shown in Equation 4:

(4) $$\vec{v_{i}}=\left(x_{i},y_{i},z_{i}\right)=\left(x_{\text{or}.}-x_{\mathrm{ins}.},y_{\text{or}.}-y_{\mathrm{ins}.},z_{\text{or}.}-z_{\mathrm{ins}.}\right),$$

where *x*, *y*, and *z* are the mediolateral, dorsoventral, and rostrocaudal components of the muscle vector; *x*_or_., *y*_or_., and *z*_or_. are the mediolateral, dorsoventral, and rostrocaudal coordinates of the muscle origin centroid; and *x*_ins_., *y*_ins_., and *z*_ins_. are the mediolateral, dorsoventral, and rostrocaudal coordinates of the muscle insertion centroid. We obtained muscle attachment centroids and areas with a modified version of the Area_Centroids_From_STL script (Davis et al., 2010; Santana et al., 2010). Details of muscle attachment site area, centroids, and force may be found in Table S3.

### 2.3 Joint reaction force calculation

To calculate JRFs, we used the reconstructed muscle forces to simulate crushing bites. The biomechanical modeling techniques are described in greater detail elsewhere (Sellers et al., 2017) but are summarized here. The computational package Boneload was used to distribute the previously calculated muscle forces over muscle attachment sites, avoiding artificial stress concentrations (Davis et al., 2010; Grosse et al., 2007). Thus, each surface node of muscle attachment sites bore some of the total muscle force. These loads were used to solve three‐dimensional finite element models in the Strand7 finite element analysis (FEA) software. A single node in the center of the articular surface of both quadrates was constrained in all three translational and all three rotational degrees of freedom, allowing us to extract components of JRF. We simulated bites both in rostral (i.e., fourth tooth) and caudal (i.e., last tooth) positions unilaterally by constraining a single node at the tip of the biting tooth in all three translational and all three rotational degrees of freedom. All muscles were modeled as contracting maximally, which is consistent with previous EMG data for crushing bites in crocodylians (Busbey, 1989; Cleuren et al., 1995).

### 2.4 Quadrate orientation and comparisons

We characterized the orientation of each quadrate using a vector that passes from the middle of the ventral articular surface to the middle of the otic condyle participating in the otic joint (Figure 1c). We refer to the axis between the dorsal and ventral contacts of the quadrate as the “structural axis” of the quadrate after Bramble (1978).

The dot product of the orientation unit vector of each quadrate and its JRF unit vector quantifies the correspondence of quadrate orientation and JRF: the degree to which quadrate orientation and JRF are co‐directional. When dealing with unit vectors, the dot product of two vectors ranges from +1 for parallel vectors to 0 for orthogonal vectors to −1 for antiparallel vectors. The calculation of a unit vector from a “typical” vector (e.g., $\vec{v_{i}}$ from Equation 4) is shown in Equation 5:

(5) $$\hat{v_{i}}=\left(\frac{x_{i}}{\left\|{\vec{v}}_{i}\right\|},\frac{y_{i}}{\left\|{\vec{v}}_{i}\right\|},\frac{z_{i}}{\left\|{\vec{v}}_{i}\right\|}\right)=\left(\frac{x_{i}}{\sqrt{{x_{i}}^{2}+{y_{i}}^{2}+{z_{i}}^{2}}},\frac{y_{i}}{\sqrt{{x_{i}}^{2}+{y_{i}}^{2}+{z_{i}}^{2}}},\frac{z_{i}}{\sqrt{{x_{i}}^{2}+{y_{i}}^{2}+{z_{i}}^{2}}}\right),$$

where $\vec{v_{i}}$ is a given vector with components $\left(x_{i},y_{i},z_{i}\right)$, and $\hat{v_{i}}$ is the unit vector of $\vec{v_{i}}$. The unit vector is a vector in which each component of the original vector is scaled by the magnitude of the original vector. These vectors have a magnitude of one. The calculation of the dot product is shown in Equation 6:

(6) $$\left\|{\hat{v}}_{Q}\cdot {\hat{v}}_{\mathrm{JRF}}\right\|=\left\|{\hat{v}}_{Q}\right\|\cdot \left\|{\hat{v}}_{\mathrm{JRF}}\right\|\cdot \cos\theta ,$$

where ${\hat{v}}_{Q}$ is the unit vector of the structural axis of the quadrate, ${\hat{v}}_{\mathrm{JRF}}$ is the unit vector of the JRF, and *θ* is the angle between the vectors. When using unit vectors, which have a magnitude of one, Equation 6 simplifies to the cosine of the angle between the two vectors, as shown in Equation 7:

(7) $$\left\|{\hat{v}}_{Q}\right\|\cdot \left\|{\hat{v}}_{\mathrm{JRF}}\right\|\cdot \cos\;\theta =1\cdot 1\cdot \cos\;\theta =\cos\;\theta .$$

**FIGURE 2:**
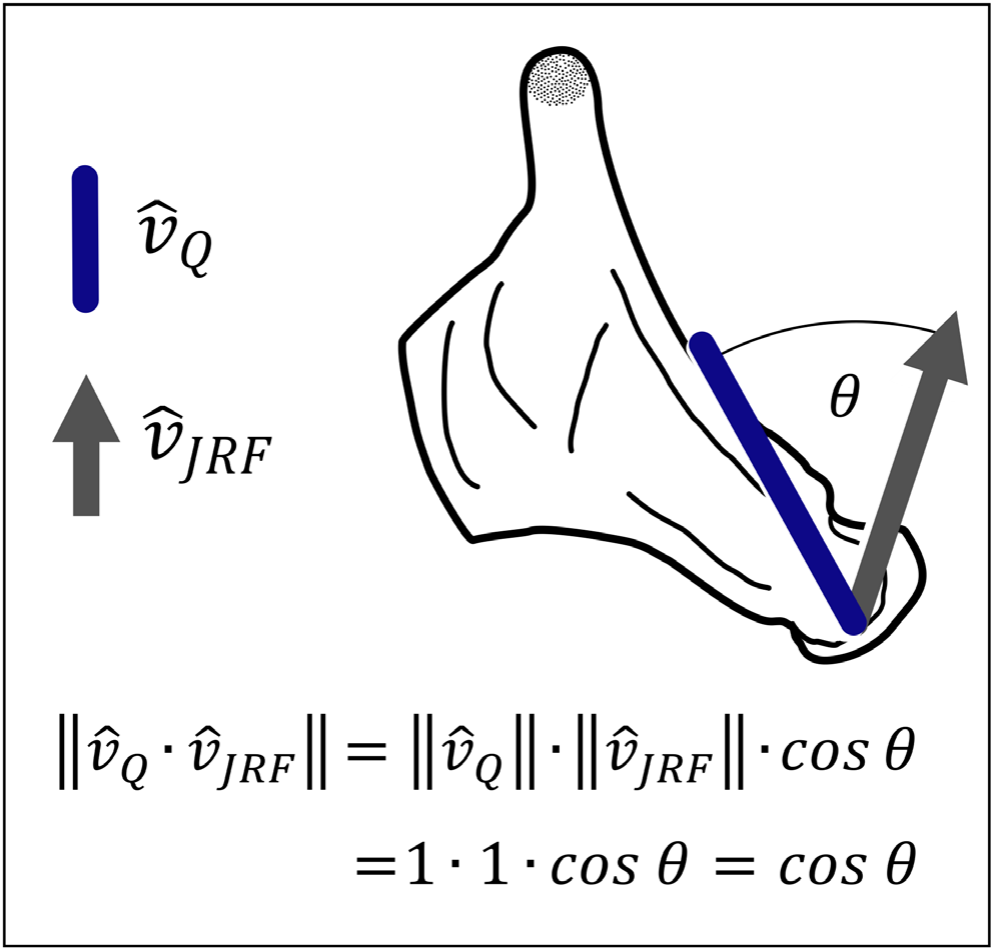
The dot product of unit vectors may be used to quantify similarity in orientation on a scale from −1 to 1. Unit vectors have a magnitude of 1 and thus characterize direction without magnitude, and allows the comparison of vectors of different units/ scales.

Thus, the dot product of two vectors represents the similarity of their orientations (Figure 2). This metric is analogous to the cosine similarity metric frequently used in data science to quantify the similarity of high‐dimensional data vectors (Salton & Buckley, 1988); here, we adopt it to quantify the similarity between three‐dimensional spatial vectors in anatomical space.

### 2.5 Quantitative analyses and hypothesis testing

All statistical analyses were conducted using the R programming language (R Core Team, 2021) in RStudio (RStudio Team, 2020) using the following packages: tidyverse (Wickham et al., 2019), dplyr (Wickham et al., 2023), reshape2 (Wickham, 2007), phytools (Revell, 2012), nlme (Pinheiro et al., 2023; Pinheiro & Bates, 2000), and ggpubr (Kassambara, 2023). Additionally, we used ggplot2 (Wickham, 2016) and ggtern (Hamilton & Ferry, 2018) for visualization. To create a distribution of vectors against which to compare our orientation data, we generated a sample of approximately 1350 unit vectors of random orientation which fill 3D space fairly uniformly. The mean dot product of this population was approximately zero, confirming that there is no bias in this sample.

We performed two analyses to test for a nonrandom relationship between the orientation of quadrates and their loading vectors (Hypothesis 1). First, for each individual, we calculated the proportion of random vectors that were a better match for the quadrate's orientation than the real JRF. We considered values below *α* = 0.05 to be a significantly nonrandom relationship. Second, we randomly sampled 11 pairs of vectors (matching our sample size) from the population of unit vectors 10,000 times. For each of the 10,000 simulated samples, we calculated the dot product of each vector pair and then calculated the sample means. We compared the mean dot product of our suchian sample against our 10,000 samples of 11 pairs of vectors. Second, we quantified the proportion of the samples that had a higher mean dot product than our suchian sample, which provides an empirical *p*‐value.

To test whether the quadrate is more suited to resist higher magnitude JRFs than lower magnitude forces (Hypothesis 2), we compared the variance in dot product for JRFs resulting from bites in caudal teeth with those resulting from bites in rostral teeth. We performed an *F*‐test to compare the variances; a significant difference in variances, with forces from rostral bites showing less variance, would support Hypothesis 2.

To test the hypothesis that the orientation of the JRF can be predicted by the orientation of the quadrate (Hypothesis 3), we performed phylogenetic generalized least squares (PGLS) regression of each component of the quadrate axis against the corresponding component of its JRF using the nlme package (Pinheiro et al., 2023; Pinheiro & Bates, 2000). A significant relationship for a given component indicates that the JRF component in that direction can be predicted by the component of the quadrate axis in that direction.

All data and code are available on Dryad (https://doi.org/10.5061/dryad.05qfttf9h); specimen models and muscle attachment sites are available on Open Science Framework (https:/osf.io/3nhmw/).

## 3 RESULTS

### 3.1 Description of matches of JRF and quadrates

**FIGURE 3:**
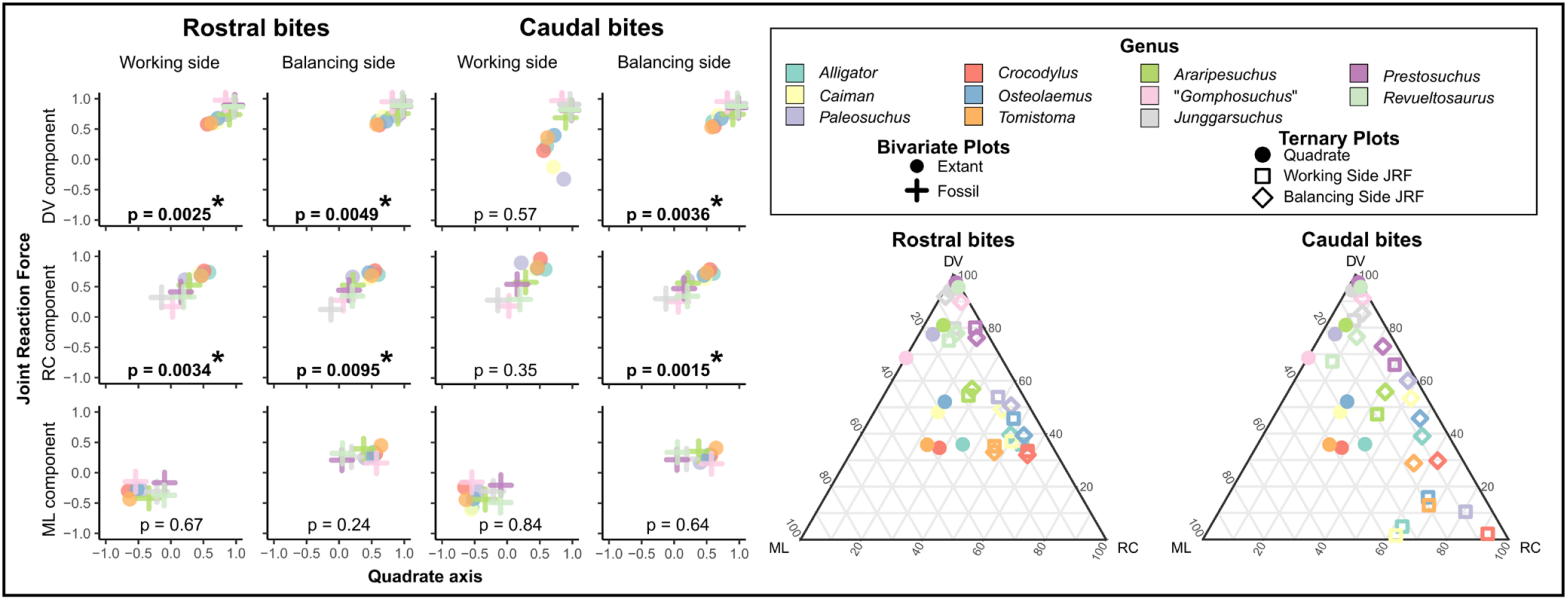
The orientation JRF can be predicted in the sagittal plane on working and balancing sides in rostral bites and on the balancing side in caudal bites. Bivariate plots showing the dorsoventral (top row), rostrocaudal (middle row), and mediolateral (bottom row) components of JRF (vertical axes) and the quadrate axis (horizontal axes) in rostral bites (left two columns) and caudal bites (right two columns) on the working side (first and third columns) and balancing side (second and fourth columns). Dorsoventral and rostrocaudal components of JRF and the quadrate axis are highly correlated, but there is no correlation between the mediolateral components. Results of PGLS are indicated on the plots; asterisks indicate significant relationships (bolded). Ternary plots confirm that JRF orientation matches the quadrate orientation more during rostral bites.

**FIGURE 4:**
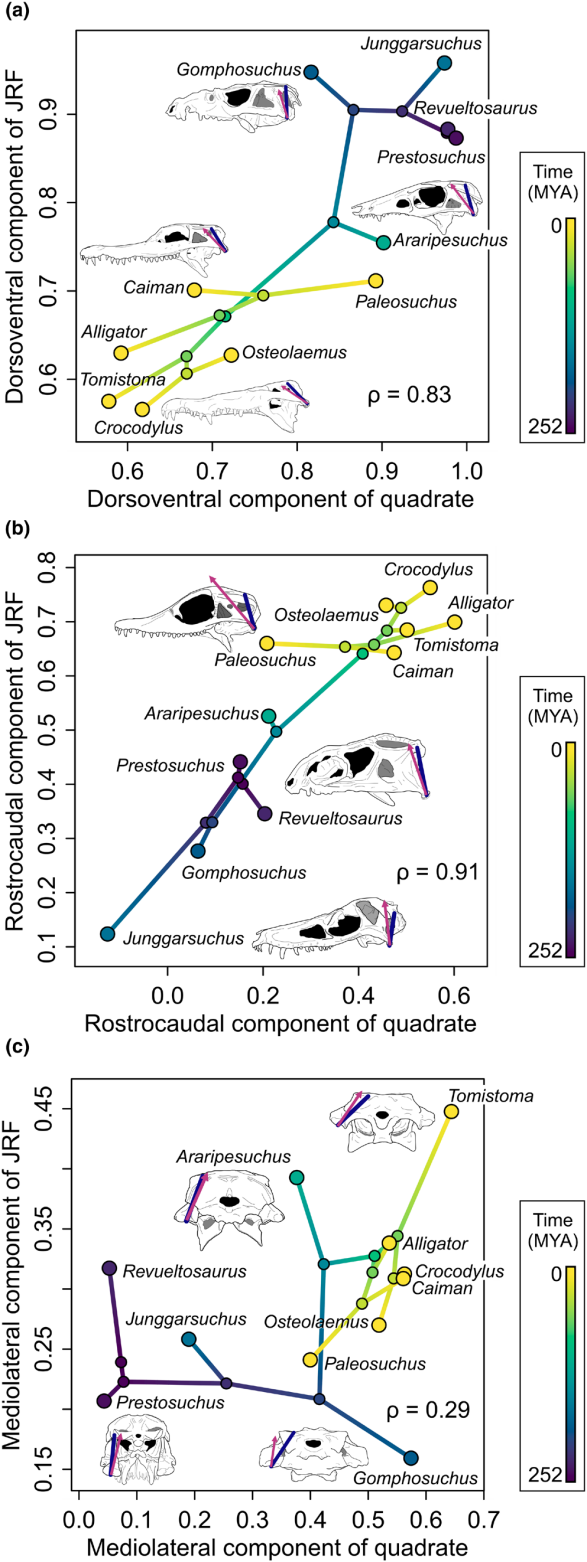
Phylomorphospaces of orientations of quadrates and JRFs demonstrate a coordinated gradual tradeoff between dorsoventral (a) and rostrocaudal (b) components as the quadrate flattened in the sagittal plane. The mediolateral component (c) of the quadrate increased as the quadrate flattened in the sagittal plane, which was followed by a later increase in the mediolateral component of JRF. Correlation coefficients of the ancestral states are in the bottom‐right of each plot. Purple line: quadrate axis. Magenta arrow: JRF.

JRF best aligned with the quadrate in rostral bites (Figures 3 and 4) but was maintained a good match with quadrate orientation during caudal bites as well. In rostral bites, the mean dot product was 0.94 (range: 0.87–0.97) on the balancing side and 0.93 (range: 0.88–0.96) on the working side. These values correspond to a mean difference of 21° (SD = 1.6°; CV = 3%), a low difference of 13° on the balancing side in *Alligator*, and high differences of 28° on the working side in *Junggarsuchus* and the balancing side of “*Gomphosuchus*.” In rostral bites, a low proportion of muscle force is transferred to the bite point; working and balancing side JRF will necessarily be more symmetrical. In caudal bites, by contrast, working side JRF is lower when bite force is higher, and this is largely driven by a reduction in the dorsal component of JRF, leading to a notable asymmetry between working and balancing side JRF. The mean dot product was 0.92 (range: 0.88–0.96) on the balancing side but only 0.78 (range: 0.045–0.93) on the working side. These correspond to a mean difference of 35° (SD = 15°; CV = 34%), a low difference of 16° on the balancing side in *Alligator*, and high differences of 87° on the working side in *Paleosuchus*. The wide range in dot products in working side JRF in caudal bites reflects the fact that the working side JRF is approaching neutral or tensile loading in some extant crocodylians, as suggested by Metzger et al. (2004).

### 3.2 Comparison of suchian quadrates with simulated vectors (Hypothesis 1)

Our comparison of suchian quadrate and JRF orientations with simulated data strongly supports Hypothesis 1 that quadrate orientation and JRF orientation are tightly coupled. For rostral bites, fewer than 5% of the simulated vectors were a better match for the quadrate orientation than the observed JRF on both sides in all taxa except the working side for *Junggarsuchus*. In caudal bites, fewer than 5% of simulated vectors were a better match in most taxa on the balancing side; fewer than 5.2% of simulated vectors were better for *Junggarsuchus*; and 5.4% were better for *Prestosuchus*. On the working side, approximately half of the tests showed values below 5%; only three taxa had values above 10%. Histograms of dot products of real quadrates with simulated vectors are available in Figure S1.

We also calculated empirical *p*‐values by comparing the mean dot product of suchian quadrates with its JRF against 10,000 samples of 11 dot products of random pairs of simulated vectors. For both working and balancing side joints in both rostral and caudal bites, no simulated vector pairs had a lower dot product (i.e., closer orientations) than the mean dot product from our observed suchian sample (*p* = 0.0001). For both working and balancing side joints in rostral bites and the balancing side joint in caudal bites, no simulated sample had a higher mean dot product than any individual suchian. For the working side joint in caudal bites, 0.03% of the samples had a higher mean dot product than *Caiman*, and 42.9% of the samples had a higher mean dot product than *Paleosuchus*. The mean and individual dot products of both bite scenarios are shown against the 10,000 simulated samples in Figure S2. The highest proportion of simulated vectors with closer orientations to the quadrate than the real joint reaction force resulting from rostral bites was found in *Junggarsuchus*; all other taxa had proportions below 5%. As hypothesized, the higher magnitude JRFs from rostral bites matched the quadrate better than the lower magnitude JRFs from rostral bites, mainly as a consequence of decreasing working side JRF magnitude.

### 3.3 Comparison of variance between rostral and caudal bites (Hypothesis 2)

Quadrate orientation matched the higher magnitude JRFs resulting from rostral bites better than the JRFs from caudal bites, supporting the hypothesis that JRF orientation is more tightly constrained when JRF magnitudes are higher (Hypothesis 2). Our analysis of variance between rostral and caudal bites showed remarkably more variance in JRF orientation in caudal bites relative to rostral bites (Zar, 2010) (*F* = 85.5, *p* < 0.0001).

### 3.4 PGLS of quadrate axis unit vector components against JRF unit vector components (Hypothesis 3)

We found partial support for the hypothesis that the orientation of the JRF can be predicted from the orientation of the quadrate (Hypothesis 3). In rostral bites with relatively symmetrical JRFs, the dorsoventral and rostrocaudal components of the quadrate axis on both the working side (*p* = 0.0025 and *p* = 0.0034, respectively) and balancing side (*p* = 0.0049 and *p* = 0.0095, respectively) predicted their respective JRF components (Figure 3). In caudal bites, variation in the working side JRF orientation means dorsoventral and rostrocaudal components are all unrelated on this side (*p* = 0.57 and *p* = 0.35, respectively), but the dorsoventral and rostrocaudal components of the balancing side quadrate predict JRF (*p* = 0.0036 and *p* = 0.0015, respectively). In no cases did the mediolateral component of the quadrate predict the mediolateral component of JRF (Rostral: *p* = 0.67 and *p* = 0.24; Caudal: *p* = 0.84 and *p* = 0.64; Figure 3). This pattern is reflected in the correlation coefficients of the estimated states of ancestral nodes (Figure 4).

## 4 DISCUSSION

Our results provide quantitative support for the hypothesis that three‐dimensional quadrate and JRF orientation are linked in tetrapods. Sinclair and Alexander (1987) estimated bite and JRF in a sample of reptiles including an individual of *Caiman*. This study estimated that the JRFs in this *Caiman* ranged from ~27° to ~67°above the horizontal, although these authors report the more vertical reaction force occurring in caudal bites rather than rostral bites. Busbey (1989) estimated joint reaction force in an *Alligator* and reported orientations ranging from ~47° to ~57° above the horizontal, although this variation was from differences in gape rather than bite location. Cleuren & de Vree (1992) also estimated joint reaction force in a *Caiman* and reported orientations ranging from ~22° to ~58° above the horizontal. Despite methodological differences, each of these studies suggested a close association between quadrate inclination and JRF orientation. Our analysis confirms these predictions and extends the results from the sagittal plane to three dimensions.

Coordination between joint structure and function is vital for jawed vertebrate evolution. As the primary site for craniomandibular interactions, the quadrate plays a crucial role in feeding. Throughout suchian evolution, the quadrate underwent correlated evolution with JRF as both transitioned from dorsoventral to oblique orientations (see Figures 3 and 4). Further, extant crocodylians have more variable JRFs (Figure 3) and thus must sometimes resist forces in suboptimal orientations. In basal suchians, the quadrate maintained the ancestral dorsoventral orientation and chondral joint (Bailleul & Holliday, 2017) with the skull roof dorsally ancestral for archosaurs (Paes Neto et al., 2021; Reyes et al., 2021). In early Crocodylomorpha, the quadrate was still largely upright, but the dorsal quadrate had migrated medially to establish contact with the braincase (Langston, 1973; Walker, 1990). By the origin of Crocodyliformes, part of the dorsal quadrate had begun to migrate rostrally by “bending” about the head of the quadrate (Busbey & Gow, 1984; Gow, 2000; Holliday & Witmer, 2009; Walker, 1990). This established the characteristic crocodyliform contact between the quadrate and the laterosphenoid (Clark, 1994; Gow, 2000; Nash, 1968; Nash, 1975; Walker, 1990). This may explain why JRF was not substantially reoriented in basal Crocodyliformes (see Figures 3 and 4). By Metasuchia, the quadrate had become less dorsally and more rostrally oriented, and JRF had reoriented to match the quadrate's oblique orientation. Further skull flattening by the origin of crocodylia resulted in an even more rostromedially oriented quadrate, this time accompanied by a further shift in JRF orientation. The correlation between the quadrate and its JRF throughout suchian evolution agrees well with hypothesized qualitative relationships between the orientation of the quadrate and its JRF or the jaw muscles (Bramble, 1978; Cleuren et al., 1995; Sinclair & Alexander, 1987). The present study tests and supports this classic hypothesis and extends it into the third (i.e., mediolateral) dimension.

The reorientation of the crocodylian quadrate has traditionally been interpreted as a mechanism of flattening the skull as part of a broader adaptation for aquatic ambush predation (Grigg & Kirshner, 2015; Iordansky, 1973). However, our results instead suggest a biomechanical explanation for the reoriented quadrate. The jaw musculature in extant crocodylians is dominated by the pterygoideus complex (Schumacher, 1973; Sellers, 2022). This muscle has an oblique orientation, with origins on the rostrum and palate. Thus, pterygoideus is not directly affected by quadrate orientation. If this transformation to jaw musculature took place as part of the flattening of the rostrum, the reorientation of the quadrate may be related to changing muscle anatomy.

Two prevailing paradigms explain how bone responds to mechanical loading. The functional matrix hypothesis states that bone form primarily develops in response to influences of surrounding soft tissues (Moss, 1962; Moss & Young, 1962). The mechanostat hypothesis states that bone is added in tissue that is excessively loaded while resorbed in areas of too little stress (Frost, 1987; Frost, 2001; Frost, 2003; Currey, 2003; Skerry, 2006). Together, these frameworks offer a basis for disentangling causal relationships between the orientation of forces and the morphology of the jaw joint. The axial component of JRF places the quadrate in compression, but perpendicular components cause bending and thus tension (Cleuren et al., 1995). Thus, two JRFs of comparable magnitudes can cause divergent loading regimes and elicit different responses in bone (Figure 1c).

**FIGURE 5:**
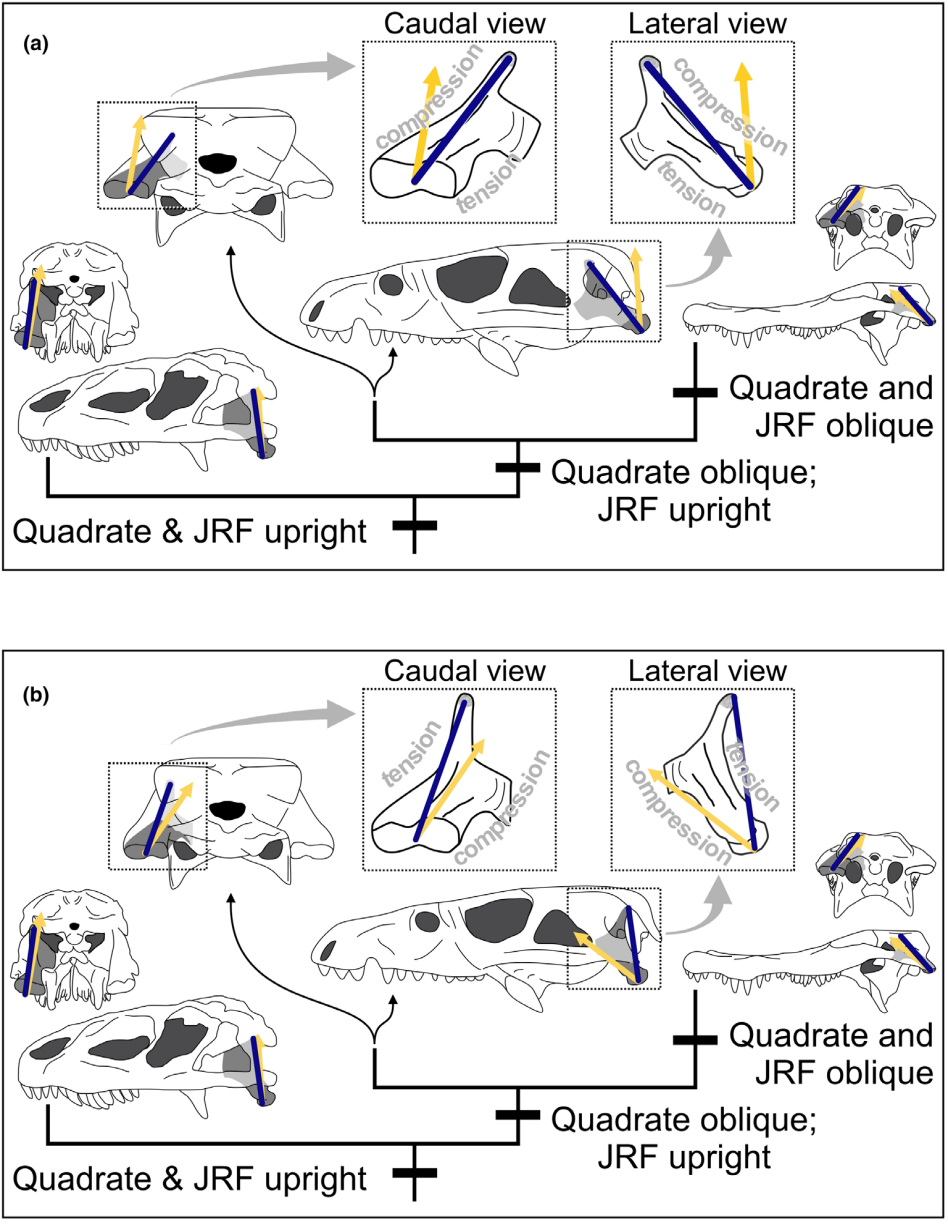
Two hypotheses for the sequence of character transitions in the evolution of the jaw joint morphology and loading. Strain patterns in intermediate forms can help us to understand the causal relationships between changing joint reaction forces (JRF; yellow arrow) and quadrate orientations (gray shading). In Scenario (a) the jaw joint reorients before JRF, leading to an intermediate condition wherein the quadrate experiences compression on its dorsocaudal aspect and tension on its ventrorostral aspect. Alternatively, in Scenario (b) JRF reorients before the jaw joint, leading to an intermediate condition wherein the loading regime is reversed.

The functional matrix and mechanostat lead to two competing hypotheses about the sequence of character transitions. If the jaw joint reoriented first as part of evolutionary (or ontogenetic) skull flattening, the perpendicular components of the dorsoventrally oriented JRF would cause compression on its dorsal aspect and tension on its ventral aspect (Figure 5a). In this scenario, muscle geometry and activity shift to bring the JRF back in line with the quadrate. Alternatively, if the JRF becomes more rostrocaudal before the jaw joint reorients, the perpendicular components of the oblique JRF would cause the opposite bending regime, with tension on its dorsal aspect and compression on its ventral aspect (Figure 5b). In this scenario, the quadrate reoriented to match the changing JRF and reduce bending. These competing scenarios of the stepwise transformations in crocodylian feeding anatomy and performance provide us with an opportunity to test hypotheses about the dynamic and coordinated relationship between ontogeny, phylogeny, the musculoskeletal system, and adaptations to cranial loading/feeding behavior during suchian evolution. Our data tentatively suggest that rotation of the quadrate in the sagittal plane was concomitant with the increasingly rostrocaudal muscle direction (Figure 4a,b), but the quadrate rotated in the transverse plane before muscles became more medially directed (Figure 4c).

**FIGURE 6:**
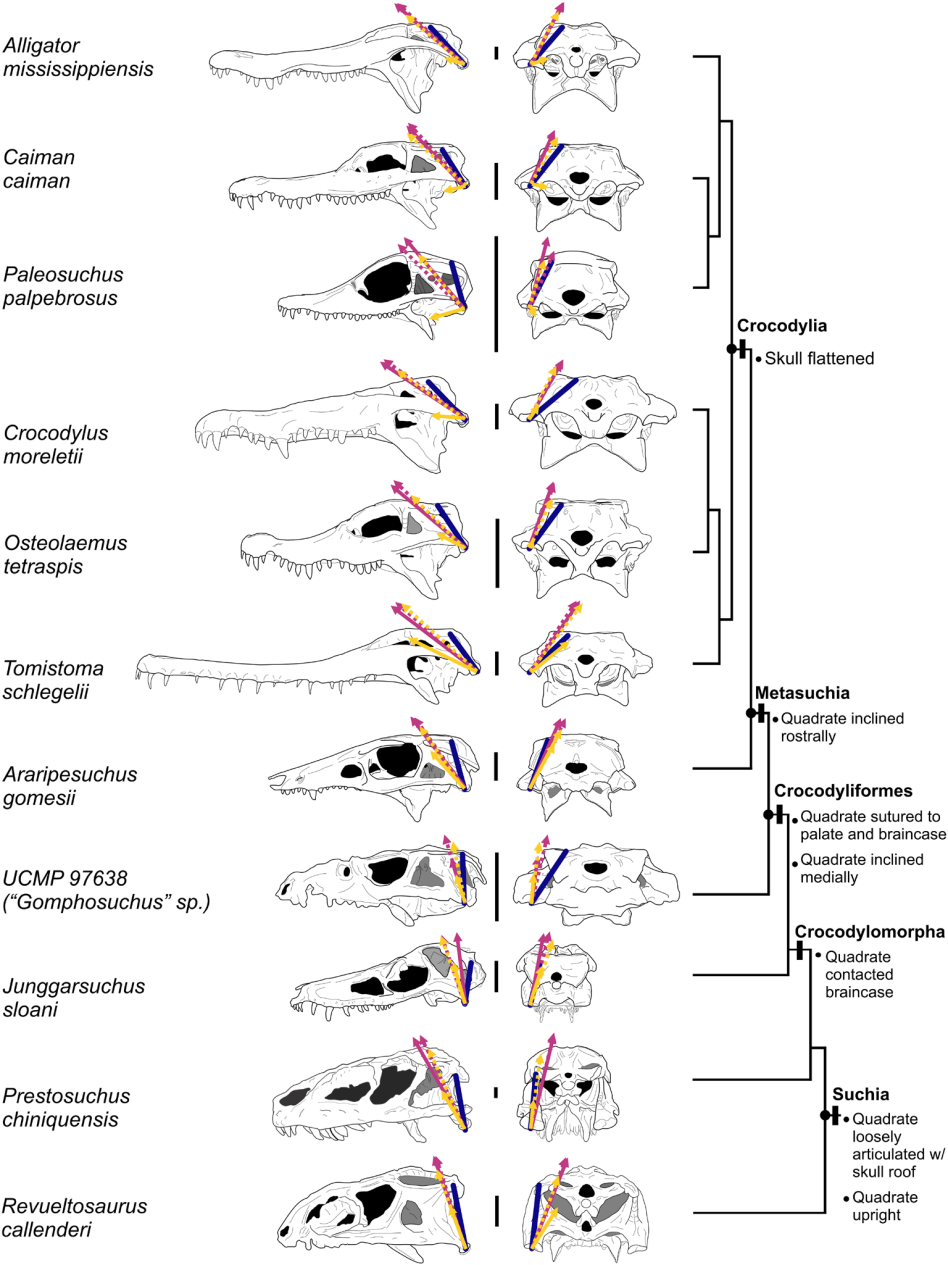
The orientation of joint reaction force becomes more flattened as the quadrate reorients in suchian evolution. Study specimens in left lateral (left) and caudal (right) views. Skulls are aligned to the left quadrate. To facilitate comparison, working side (solid arrows) and balancing side (dashed arrows) JRFs from rostral bites (magenta arrows) and caudal bites (yellow arrows) are plotted on the left quadrate along with the “structural axis of the quadrate” (dark purple line). All scale bars are 2.5 cm.

Crocodylians are characterized by a suite of derived cranial features traditionally associated with resisting high bite force (Figure 6). Features such as a robust rostrum with scarf joints and an extensive secondary palate, associated with the aquatic ambush predatory phenotype, have clear roles in resisting the powerful dorsoventral bending that accompanies powerful biting in a long, flat rostrum (Busbey, 1995). Other modifications such as the sutural union of the quadrate and palate with the braincase and the accompanying loss of cranial kinesis were made earlier in the lineage—before the acquisition of aquatic ambush predation in the lineage leading to crocodylia (Busbey, 1995; Clark et al., 2004; Holliday & Witmer, 2008, 2009; Iordansky, 1973; Langston, 1973; Wilberg et al., 2019). These modifications are not associated with the rostrum but are instead involved in resisting JRFs. The present study suggests that animals near the base of crocodyliformes may be in a rare state of mismatch between quadrate orientation and JRF. In crania with a mobile otic joint, a JRF that does not pass through the joint will rotate the quadrate, potentially causing unexpected shifts in the kinetic apparatus (Sinclair & Alexander, 1987). Thus, the consolidation of the crocodyliform cranium and loss of akinesis may represent an adaptation to oblique joint forces and serve as a preadaptation for rather than adaptation to generating high bite forces with a flat skull.

## 5 CONCLUSION

Here, we show how the quadrate and JRF coevolved during the characteristic apomorphic flattening of the skull during crocodylian and suchian evolution. We explored the relationship between quadrate morphology and loading, highlighting the importance of the quadrate as the primary site of movement and force transfer between the cranium and mandible in nonmammalian tetrapods. We use biomechanical modeling to show that quadrate orientation and JRF orientation are tightly coupled during evolution. We also found support for the hypothesis that the quadrate is more suited to higher magnitude forces than lower magnitude forces, suggesting that selection acts to align the quadrate with the greatest forces it experiences. Finally, we found partial support for the hypothesis that we can predict JRF orientation from quadrate orientation. These lines of evidence demonstrate a coordinated evolution between anatomical and functional aspects of the jaw joint in one of the great transformations in vertebrate evolution. Overall, this study advances our understanding of the functional morphology and biomechanics of nonmammalian tetrapod craniomandibular joints and provides a foundation for exploring similar evolutionary transformations in other lineages.

## AUTHOR CONTRIBUTIONS

**Kaleb Sellers**: conceptualization, data curation, formal analysis, funding acquisition, investigation, methodology, software, resources, visualization, writing—original draft, and writing—review and editing. **Alec Wilken**: software and visualization. **Corrine Cranor**: data curation and visualization. **Kevin Middleton**: conceptualization, investigation, methodology, resources, and software. **Casey Holliday**: conceptualization, data curation, funding acquisition, investigation, methodology, resources, writing—original draft, and writing—review and editing.

## Supporting information


Data S1.

**Table S1.** Details of finite element model construction.

**Table S2.** Links to Sketchfab models of suchian crania, mandibles, and attachment sites.

**Table S3.** Parameters used to estimate muscle force and load finite element models.

## Data Availability

All models, data, and code used for analyses are available on Dryad (https://doi.org/10.5061/dryad.05qfttf9h). Specimen models and muscle attachment sites can be visualized on Sketchfab (https://sketchfab.com/holliday/collections/suchian‐jaw‐muscle‐attachments‐98f1919fdbe84e0eba7e1264b919cc28).
