# Supplementary material for: Quadrate orientation and joint reaction force underwent correlated evolution during suchian evolution
Source: J Anat. 2025 Jul 22;248(6):890–901. doi: 10.1111/joa.70020 (PMC13148634; doi:10.1111/joa.70020)

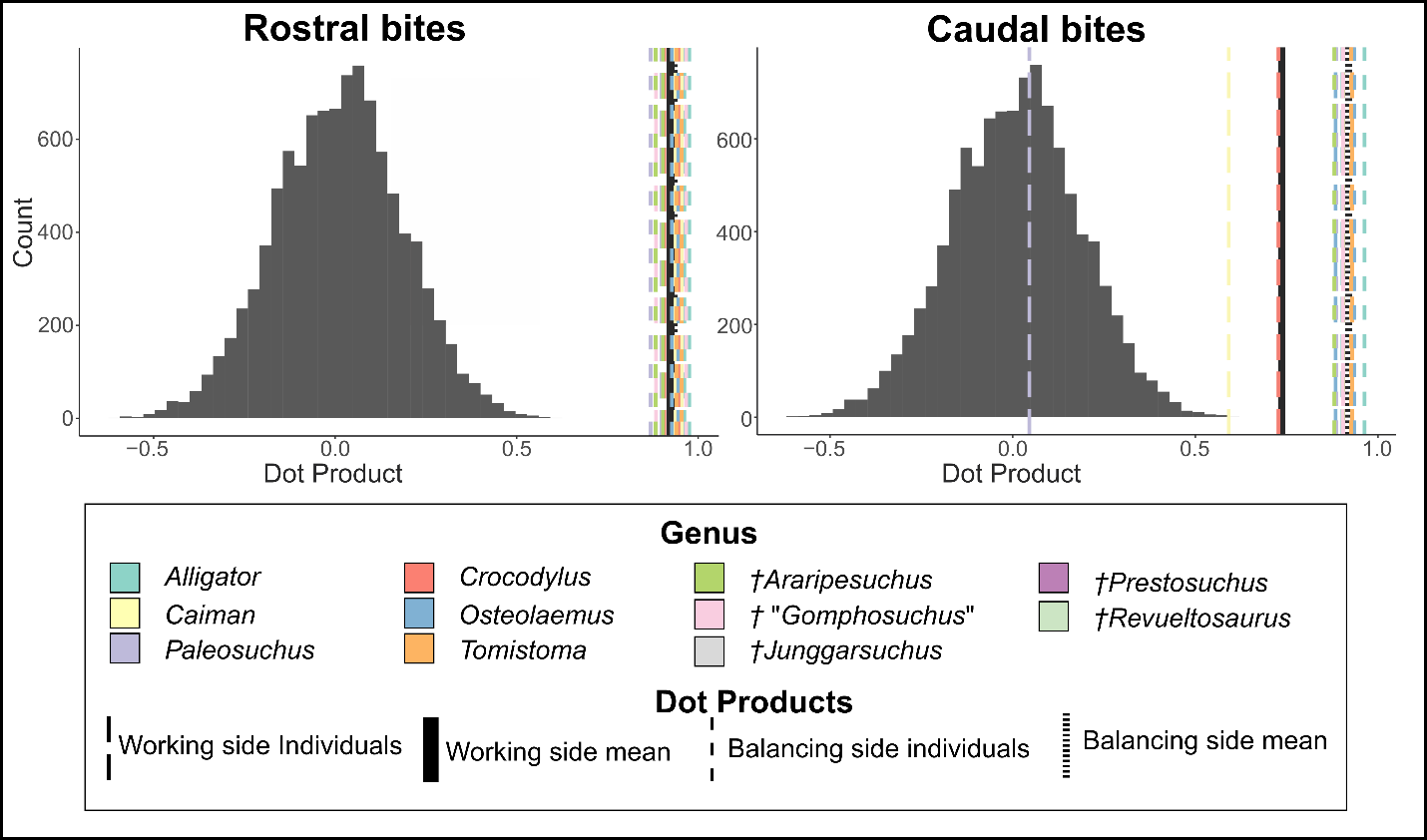
Figure S1. Quadrates and their JRFs are closer in orientation than chance would predict, and the match is closer when JRF is higher. Histograms show distribution of mean dot products of 10,000 samples of 11 random vectors and individual and mean suchian dot products. Mean dot product of quadrate axes and JRFs was higher for both working and balancing side for rostral and caudal bites, as were most individual suchian dot products, but in caudal bites, two alligatoroids (*Paleosuchus* and *Caiman*) had dot products below some simulated samples. This results from the predicted tensile loading of working-side joints in these taxa.


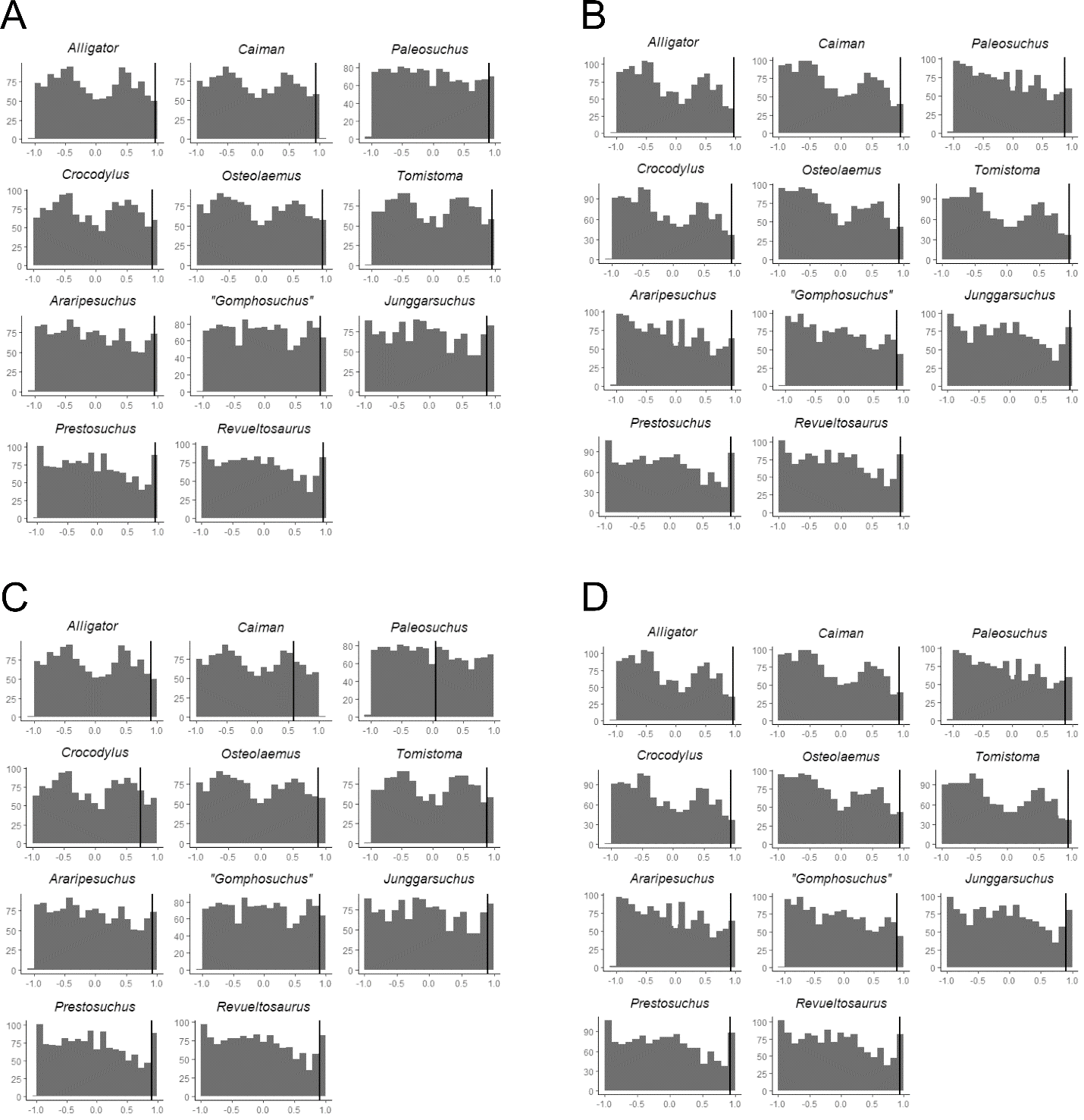
Figure S2. Histograms of dot products of ~1350 random vectors and quadrate axes. Horizontal axes are dot products and vertical axes are counts. Vertical line indicates dot product of real JRF and quadrate orientation. A) Working side in rostral bites, B) Balancing side in rostral bites, C) Working side in caudal bites, D) Balancing side in caudal bites.


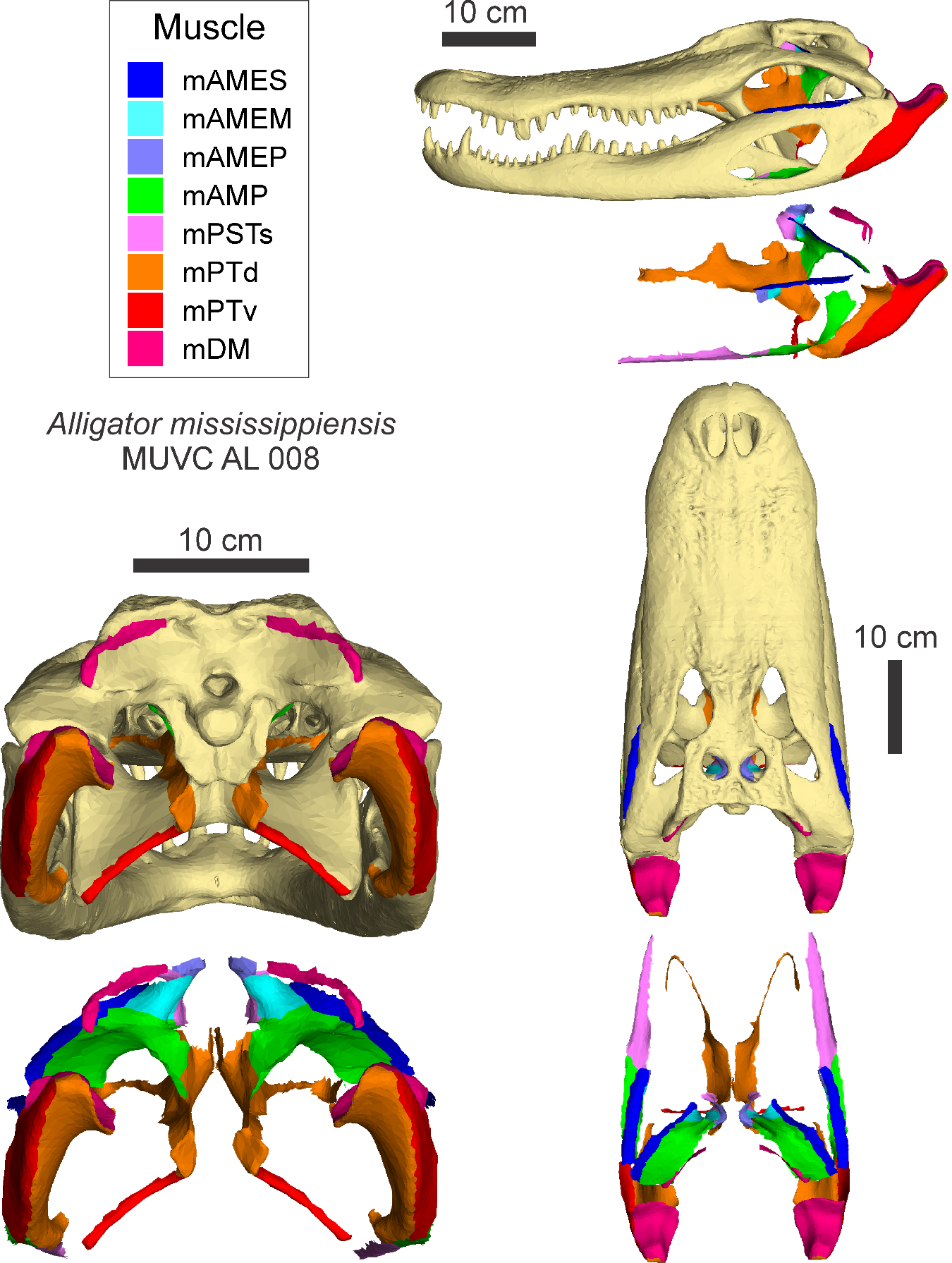
Figure S3. Muscle attachments in *Alligator mississippiensis*. Top-left, muscle homology color scheme. Top-right, left lateral view. Bottom-left, caudal view. Bottom-right, dorsal view.

Figure S4. Muscle attachments in *Caiman crocodilus*. Top-left, muscle homology color scheme. Top-right, left lateral view. Bottom-left, caudal view. Bottom-right, dorsal view.


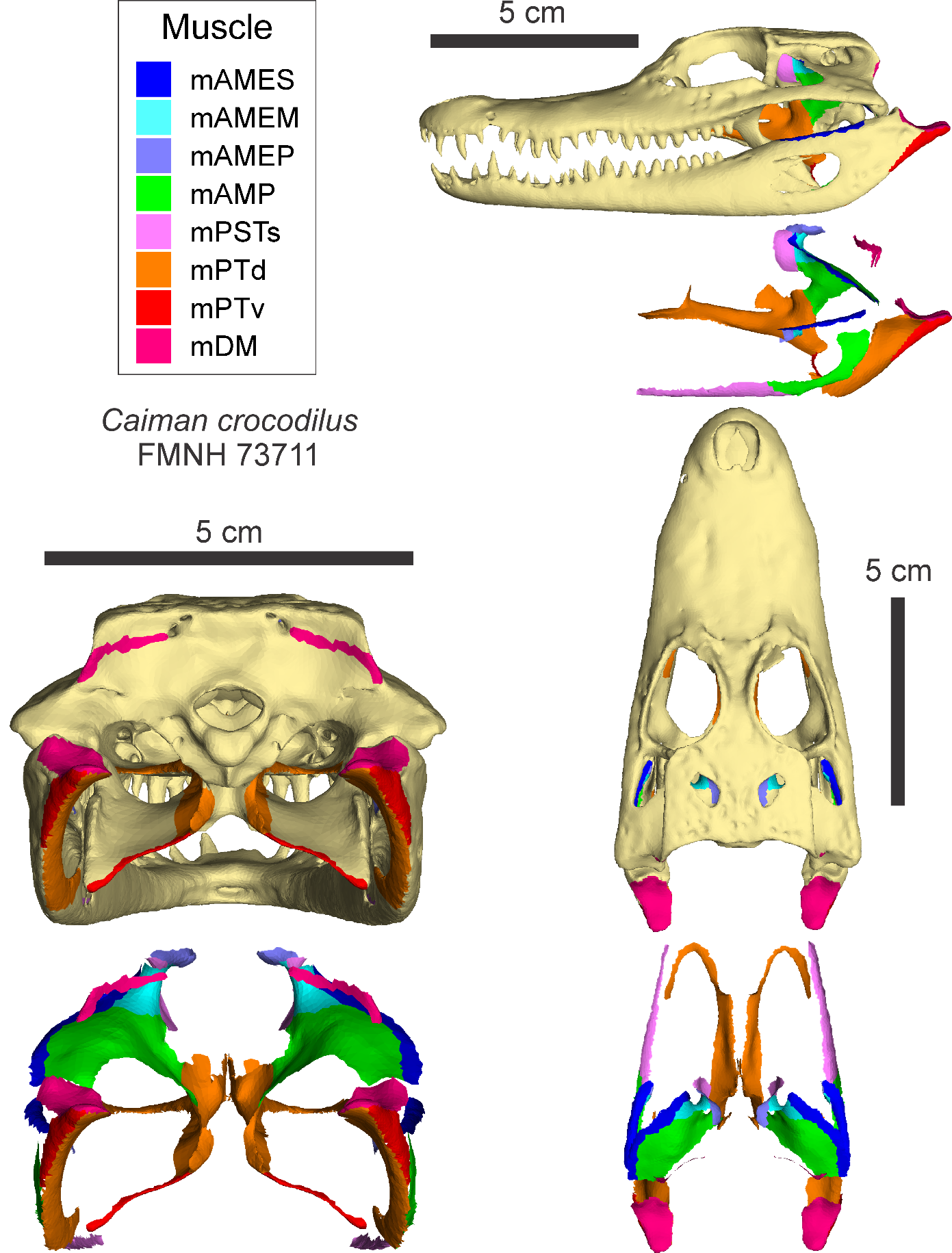


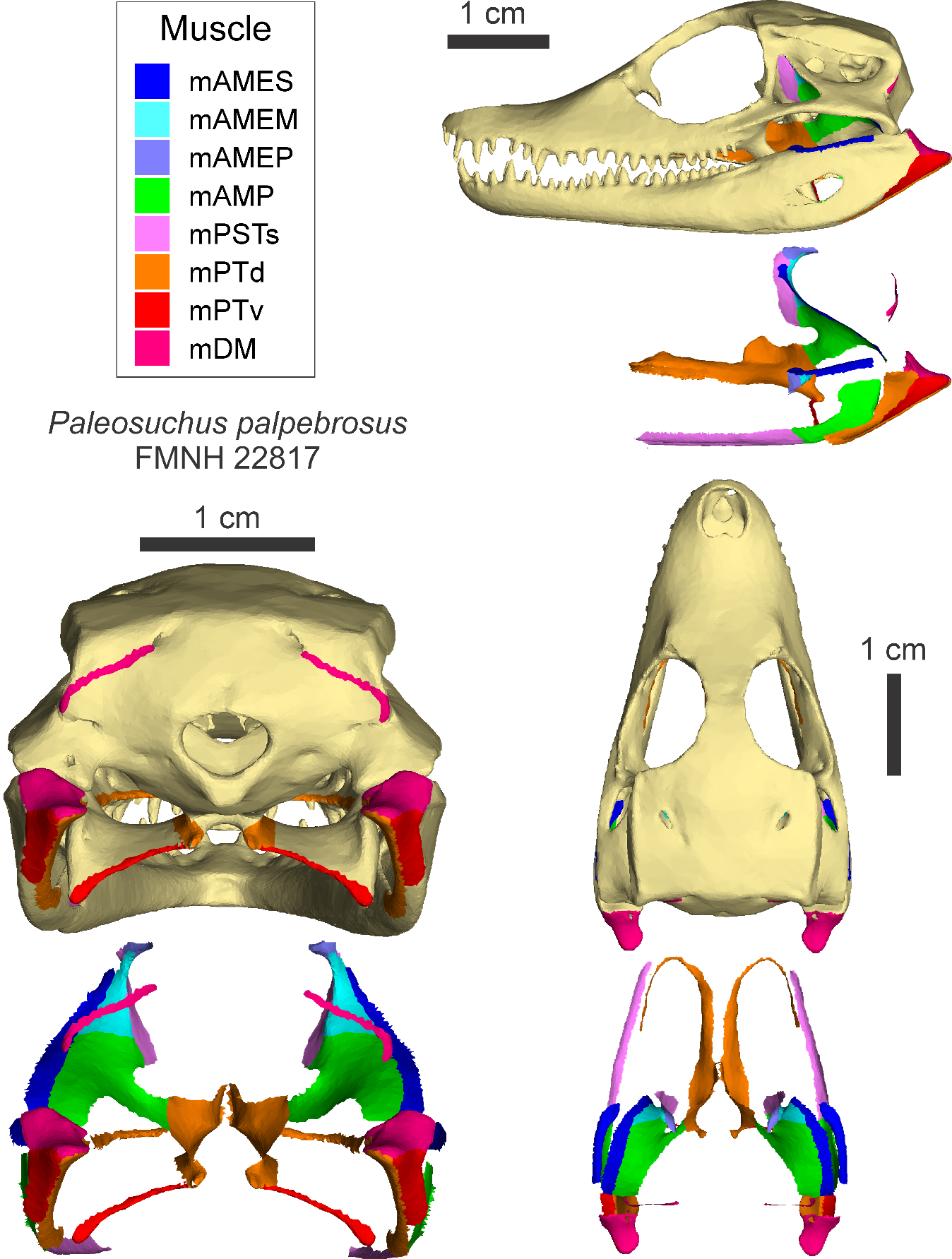
Figure S5. Muscle attachments in *Paleosuchus palpebrosus*. Top-left, muscle homology color scheme. Top-right, left lateral view. Bottom-left, caudal view. Bottom-right, dorsal view.

Figure S6. Muscle attachments in *Crocodylus moreletii*. Top-left, muscle homology color scheme. Top-right, left lateral view. Bottom-left, caudal view. Bottom-right, dorsal view.


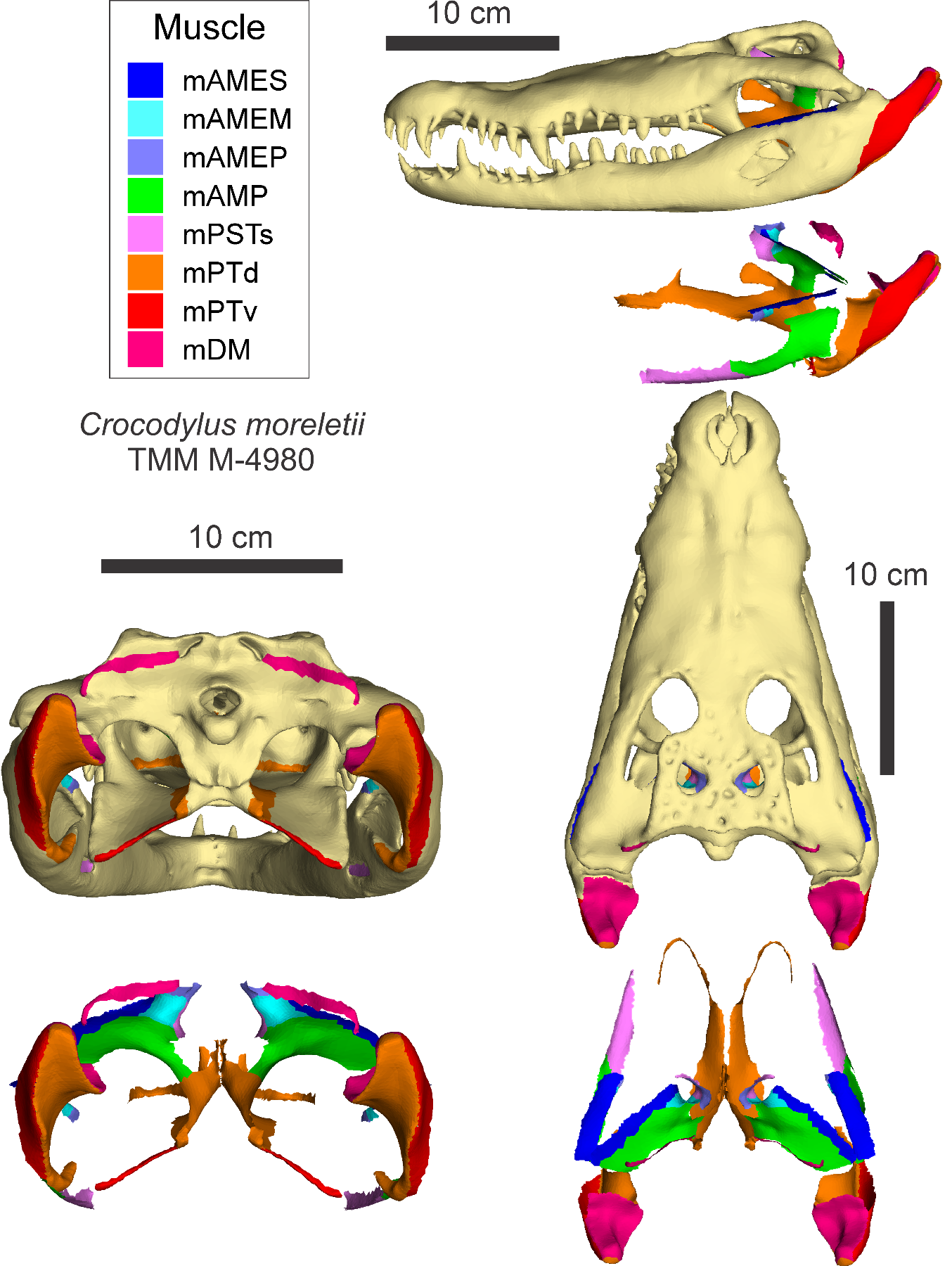


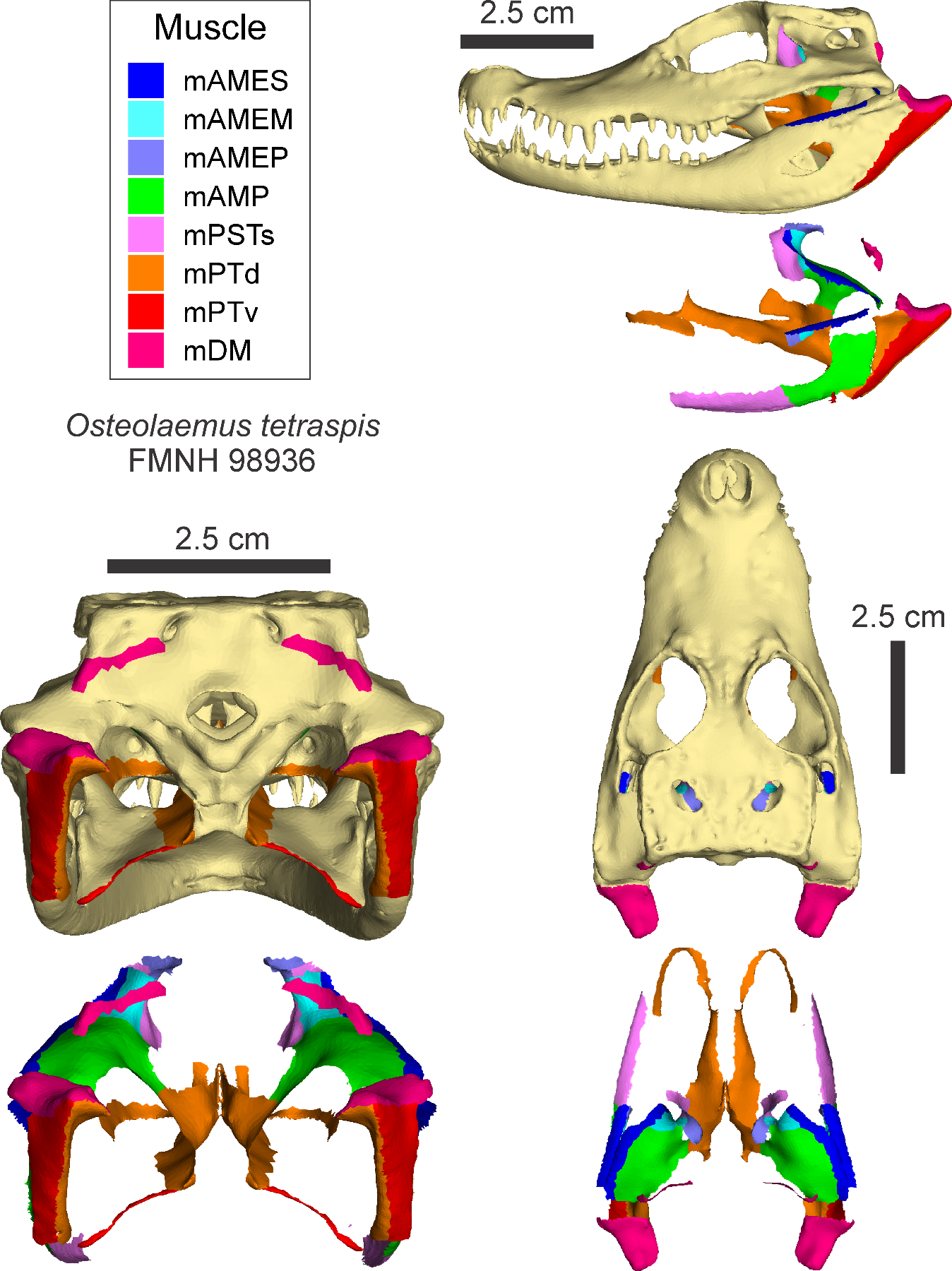
Figure S7. Muscle attachments in *Osteolaemus tetraspis*. Top-left, muscle homology color scheme. Top-right, left lateral view. Bottom-left, caudal view. Bottom-right, dorsal view.


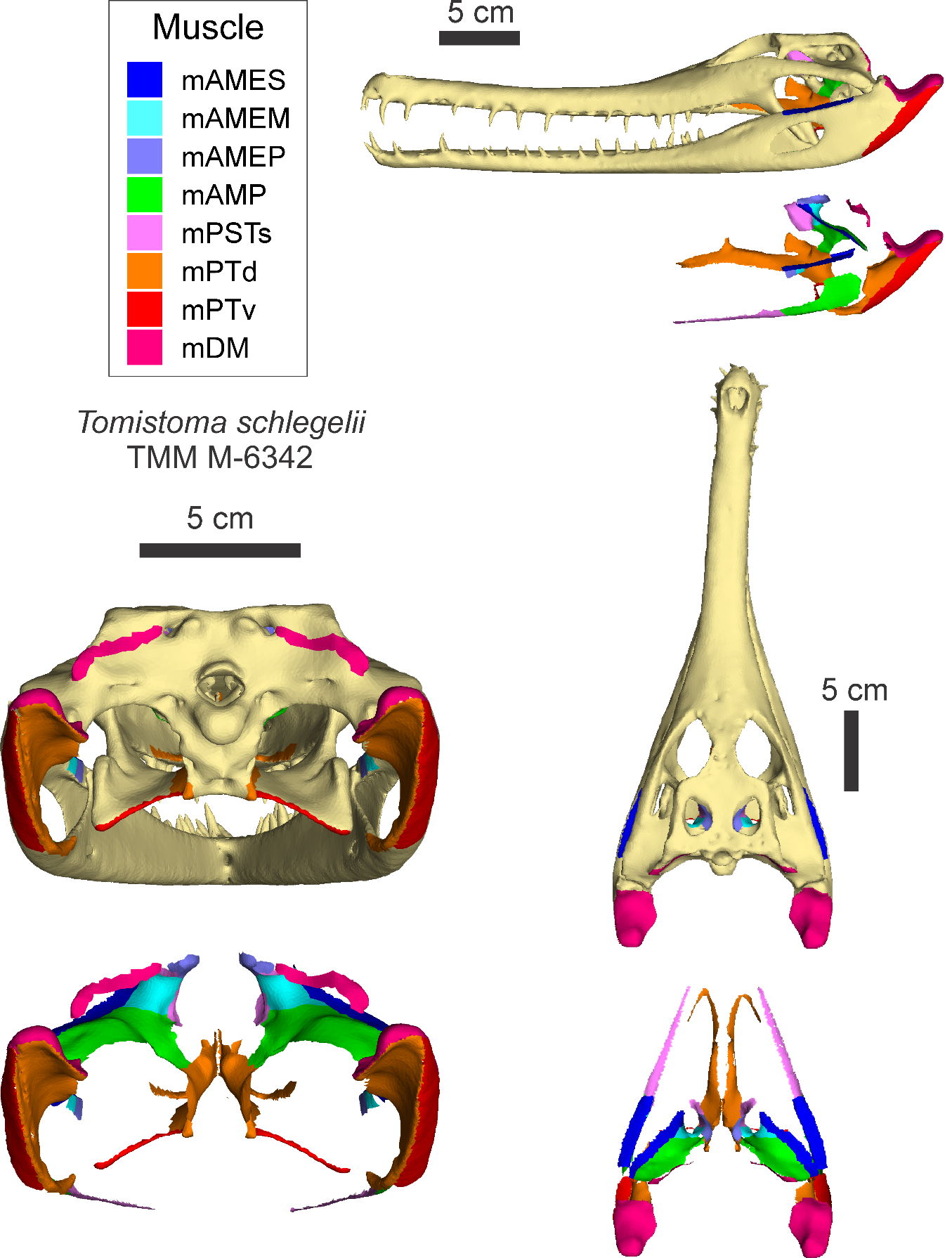
Figure S8. Muscle attachments in *Tomistoma schlegelii*. Top-left, muscle homology color scheme. Top-right, left lateral view. Bottom-left, caudal view. Bottom-right, dorsal view.


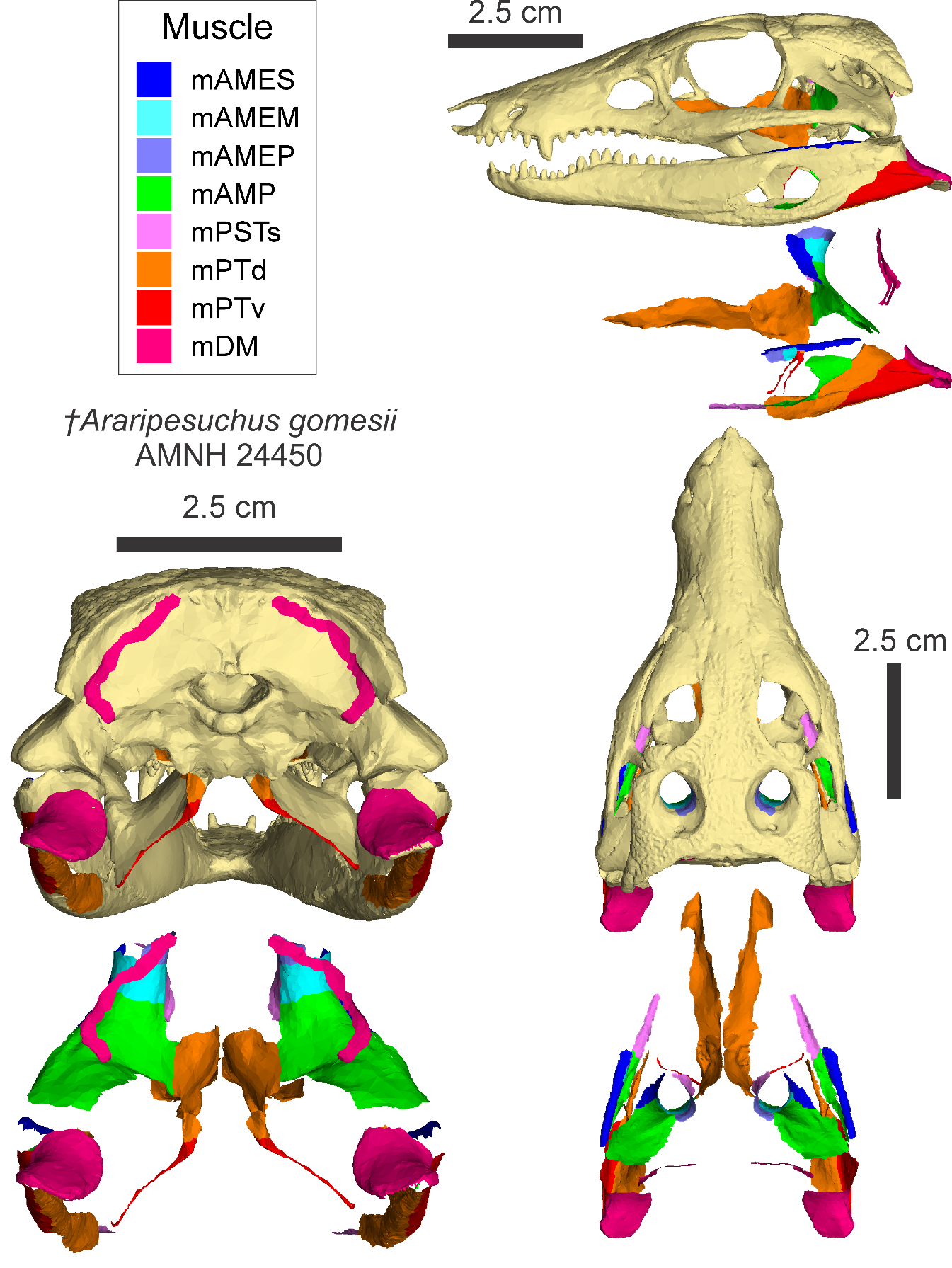
Figure S9. Muscle attachments in *†Araripesuchus gomesii*. Top-left, muscle homology color scheme. Top-right, left lateral view. Bottom-left, caudal view. Bottom-right, dorsal view.


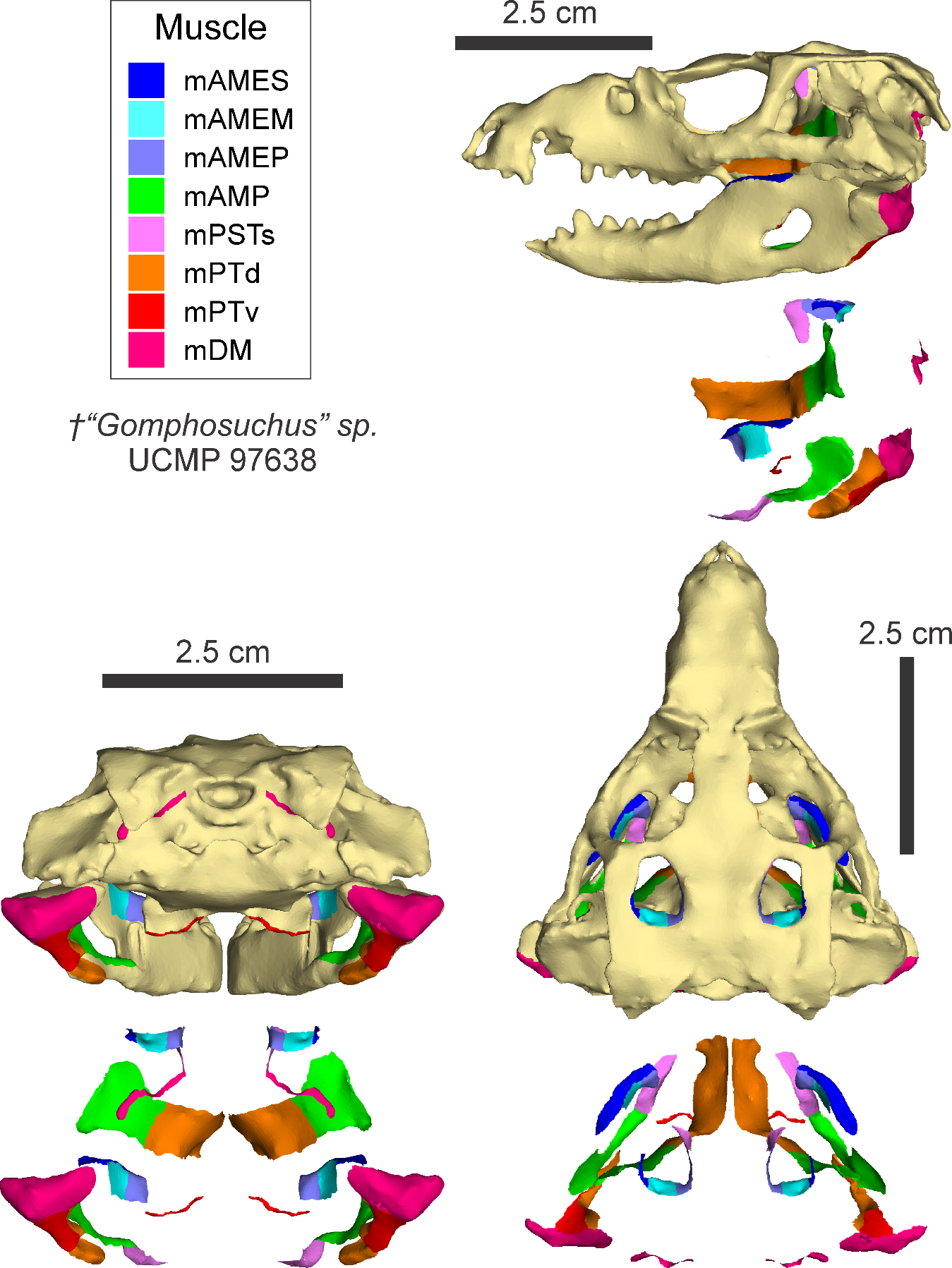
Figure S10. Muscle attachments in *†*“*Gomphosuchus* sp.” Top-left, muscle homology color scheme. Top-right, left lateral view. Bottom-left, caudal view. Bottom-right, dorsal view.


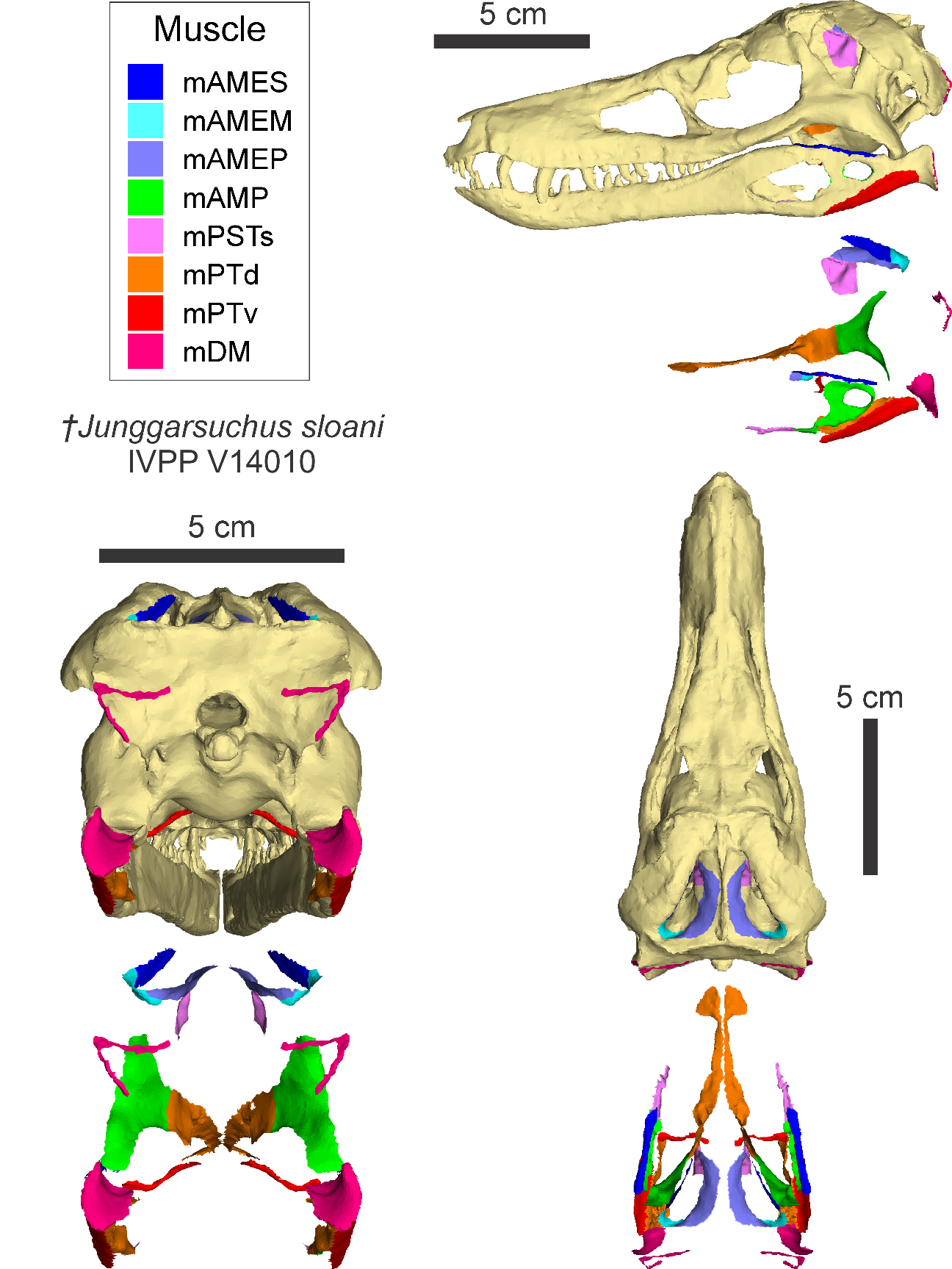
Figure S11. Muscle attachments in *†Junggarsuchus sloani*. Top-left, muscle homology color scheme. Top-right, left lateral view. Bottom-left, caudal view. Bottom-right, dorsal view.


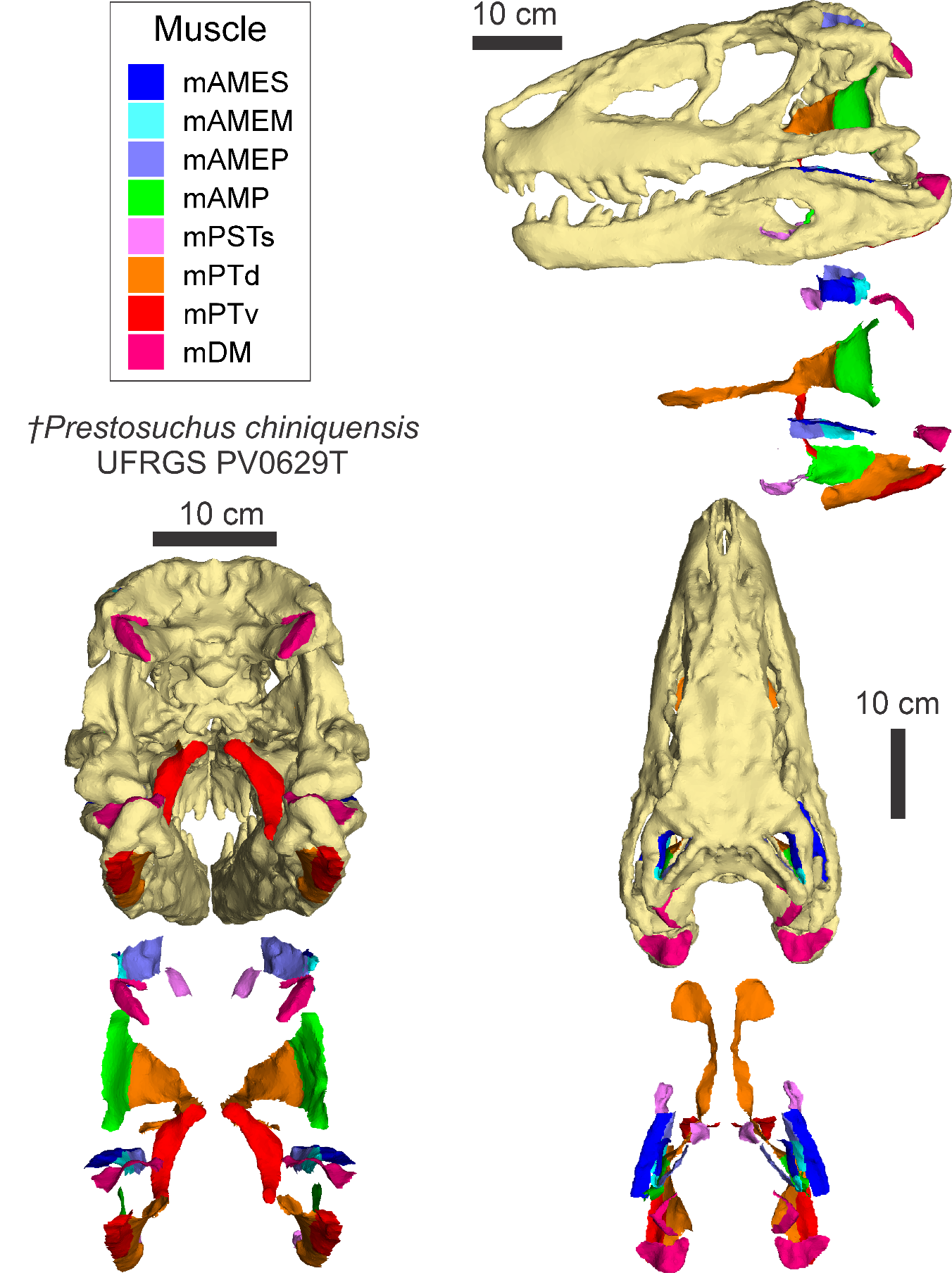
Figure S12. Muscle attachments in *†Prestosuchus chiniquensis*. Top-left, muscle homology color scheme. Top-right, left lateral view. Bottom-left, caudal view. Bottom-right, dorsal view.

Figure S13. Muscle attachments in *†Revueltosaurus callenderi*. Top-left, muscle homology color scheme. Top-right, left lateral view. Bottom-left, caudal view. Bottom-right, dorsal view.


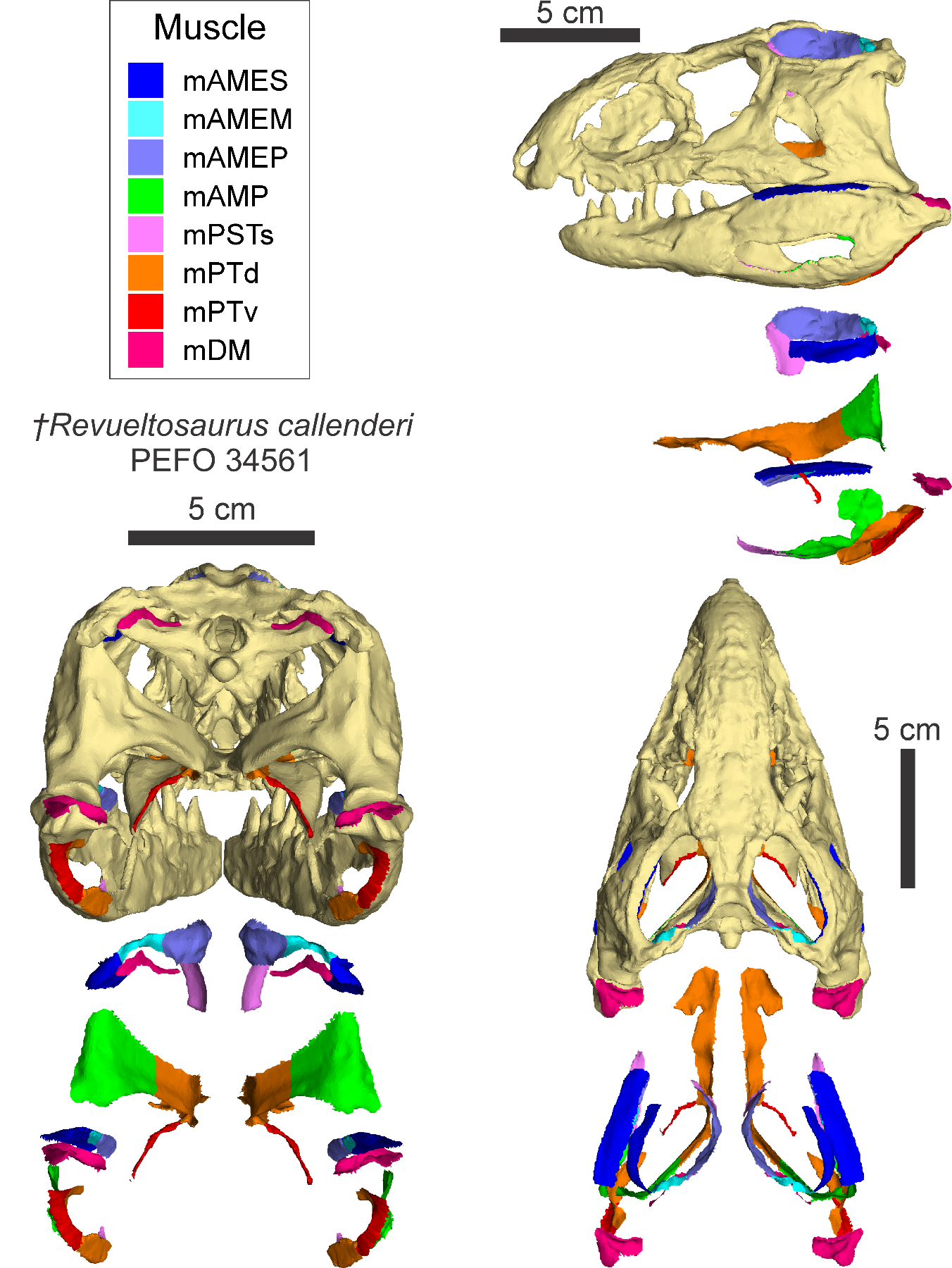

Supplement: Supplementary file 1 — Data S1. [file JOA-248-890-s001.docx]
